# Dialysed Iron

**Published:** 1878-01-20

**Authors:** 


DIALYZED IRON.—This article is of recent introduction, and is attracting very favorable attention as being perhaps the best chalybeate which we have. It agrees well with the stomach, is not unpleasant to the taste, and does not constipate the bowels. It is said to be antidotal to arsenic, a fact which the profession will gladly hail. A case illustrative of its property in this respect may be found among the selected articles in to-day's journal. The article used was that prepared by John Wyeth & Bro., Philadelphia.

We have for sometime been using this same preparation as a tonic and chalybeat with very satisfactory results.

